# Towards an understanding of inequalities in accessing residential and nursing home provision: The role of geographical approaches

**DOI:** 10.1111/hsc.13770

**Published:** 2022-02-25

**Authors:** Gary Higgs, Mitchel Langford, Mark Llewellyn

**Affiliations:** ^1^ 6654 Faculty of Computing, Engineering and Science Wales Institute of Social and Economic Research and Data (WISERD) and GIS Research Centre University of South Wales Pontypridd UK; ^2^ 6654 Welsh Institute for Health and Social Care University of South Wales Pontypridd UK

**Keywords:** accessibility, COVID‐19, residential and nursing beds, social care, spatial inequalities, Wales

## Abstract

Suggestions of the existence of so‐called ‘social care deserts’ in England in the years leading up to the COVID‐19 pandemic drew attention to the potential impact of geographical inequalities on the availability of residential, nursing and domiciliary care. To date, much of this analysis has been conducted at spatially aggregated scales such as that of local authorities or postcode sector. Hidden within such aggregate‐level analysis however are geographical differences in the local provision of care services. In this paper, we draw attention to geographical modelling techniques that can be used to examine local trends in the supply of social care services in relation to potential demand. These spatial models can be used to examine variations in the number of facilities (or choice) within reasonable drive times/distances. Drawing on a national database of residential and nursing care beds in Wales for March 2020, we illustrate the potential of such techniques to provide an insight into current patterns in access to care homes, and to monitor future changes in the fall‐out from the effects of the COVID‐19 pandemic on the care home sector. The concentration of care home sites in metropolitan areas and in the heavily populated post‐industrial valleys in the south‐east is identified, but significant demand present in these areas ameliorates scores towards mid‐range ratios. We conclude by suggesting that the types of techniques used in this study enable disparities in provision within localised areas to be better explored, thereby helping planners and policy makers to address potential inequalities in provision.

**What is known about this topic**

- Prior to the COVID‐19 pandemic, there were concerns surrounding so‐called ‘care deserts’ with some postcode sectors having a lack of residential and nursing care places.
- To date, studies of geographical access have tended to be at relatively coarse, aggregate scales which do not permit an analysis of localised patterns of provision.
- The impacts of COVID‐19 on the care home sector has drawn attention to the need to monitor the potential impacts of the pandemic on such provision.

**What this paper adds**

- Traditional measures of access are critiqued before techniques are described which examine variations in supply in relation to potential demand for places.
- Benchmark maps of access have been developed which demonstrate how the impacts of the pandemic can be examined at detailed spatial scales.
- The study has drawn attention to existing inequalities in access to residential and nursing care places that can help local authorities in planning the provision of social care.

## 1 INTRODUCTION

The coronavirus pandemic has more than ever raised the media spotlight on the high rates of incidence and mortality attributed to COVID‐19 within residential and nursing care homes both in the UK (Daly, 2020; Hollinghurst et al., 2021) and in countries such as Canada (Molinari & Pratt, 2021), France (Belmin et al., 2021) and Italy (Ventura et al., 2021). This has drawn attention to the need to understand immediate and longer‐term impacts of the pandemic on the care home sector. This study considers how to monitor impacts on the availability of residential and nursing care places at localised level using geographical approaches to modelling patterns of provision.

Prior to the onset of the pandemic, concerns were expressed regarding overall provision of places within residential and nursing care homes at a time of raised demand stemming from an increasingly aged population and a projected increase in the prevalence of multi‐morbidity within such age cohorts (Kingston et al., 2018). In the UK context, this may be compounded by trends in nursing care provision and a lack of capacity caused by providers shifting from nursing to residential care due to shortages of people with appropriate nursing skills and qualifications in some settings. Such trends may have been exacerbated COVID‐19 and potential tightening of local authority budgets in subsequent years. Prior to the pandemic an Age UK commissioned report examined spatial patterns in availability of care home and nursing home beds at postcode sector level in England (Incisive Health, 2019). Their contention of so‐called ‘care deserts’ or a ‘geographical lottery’, based on the availability of beds per 1000 population aged over 65, also highlighted the need for more detailed consideration of geographical patterns of capacity in relation to potential demand for provision.

The aims of this paper are firstly to provide a cross‐sectional analysis of residential and nursing care availability across Wales at small area level (as of March 2020) and, secondly, to draw upon this analysis to examine the wider utility of techniques that may be further enhanced by the incorporation of data on for example the nature and quality of provision in care homes. In Wales, as across the UK, care provision is a responsibility of local authorities who engage in commissioning exercises to ensure residential and nursing care places are provided within their localities, conducting needs assessment exercises to ensure a sufficient quantity and quality of provision (Institute of Public Care, 2017; Public Policy Institute of Wales, 2015). Under the Social Services and Well‐being (Wales) Act (2014), Regional Partnership Boards (RPBs) are charged with developing area plans based on local population needs assessments at regional level to help ensure care homes are local and accessible for those needing residential placements (National Assembly for Wales, 2014). However, publicly as well as privately funded people who live in Wales are also able to choose a care home in England (and vice versa).

A consumer research exercise conducted by Ipsos MORI (2017) drew attention to the types of factors influencing the choice and eventual take‐up of places for residents admitted to care homes in the UK. Whilst these included factors such as the quality of care, the suitability of the care home to the person's needs, care home fees and culture of the care home (including whether the home cares for people from a particular faith or ethnic community) for many the imperative is to minimise the distance from the resident's current home address to facilities that are convenient for visiting friends and relatives. The findings of a Competition and Marketing Authority (CMA) report (2017; p.36) suggest that ‘location is the main factor for potential residents and their representatives when choosing a care home’ which in turn draws attention to the importance of the familiarity offered by local provision, the use of convenient transport options and the need to limit travel for those visiting care homes. Given the evident significance of location, an important contribution of this study relates to the development of techniques that enable patterns of access in residential and nursing care provision to be revealed. This may be particularly pertinent given the pressures likely to arise on the care home sector in the aftermath of the COVID‐19 pandemic and addresses the need for a more detailed analysis of the availability of care home places in relation to anticipated patterns of demand.

## 2 GEOGRAPHICAL APPROACHES TO EXAMINE INEQUALITIES IN ACCESS TO RESIDENTIAL AND NURSING CARE HOMES

### 2.1 Investigating spatial patterns of residential and nursing care home provision

Previous studies have considered accessibility to services such as libraries, transport, health and social services (Fransen et al., 2015; Horner et al., 2015; Lange & Norman, 2018; Lin et al., 2014), but few examples exist in published literature on exploiting Geographical Information System (GIS) to measure access to residential and nursing care homes and other social care provision for the elderly. Early studies that focused on geographical aspects of different forms of nursing, residential care and support services for the elderly were conducted at relatively coarse regional or national scales (Andrews & Phillips, 2002; Corden, 1992; Ford & Smith, 1995, 2008; Hamnett & Mullings, 1992; Phillips & Vincent, 1986; Phillips et al., 1987; Smith & Ford, 1998). Others have considered the wider implications of individual or group care home closures (e.g. Glasby et al., 2019; Netten et al., 2005). Concerns regarding changing levels of social care supply in relation to demographic trends are also mirrored in wider international contexts (see for example Fret et al., 2019; Matei et al., 2018; Walker et al., 2019).

Only recently have more advanced spatial analytical approaches been used to analyse and visualise variations in accessibility to residential or community care provision for the elderly at more detailed spatial scales (Chotvijit et al., 2018; Frochen et al., 2019, 2020; Jia et al., 2018; Kilinc et al., 2017; Liu et al., 2020; Matei et al., 2018; Vrabková et al., 2021). These have primarily used either a proximity or a density measure to investigate service variations. Proximity measures usually identify the nearest facility from modelled or estimated demand points using either straight‐line or network distance/travel time. In contrast, density metrics are area‐based and record the number of facilities in each administrative area or census tract standardised by a population count such as those aged 70 years or more, to yield a provider‐to‐population ratio. An example of this approach was included in the Competition and Markets Authority (2017) report (p. 36) which defined a local market by a 15 to 20 drive time which they equated as representing three, five and 10 miles respectively for urban, suburban and rural areas. Accordingly, in their analysis of home care provision in England, Scotland and Wales at the postcode sector level, they used drive time isochrones to examine what percentage of such sectors had three or more different providers within a 15‐min drive time of the centre of each sector. Overall, 90% of all postcode sectors had this level of provision with 19% having two or fewer predominantly located in rural areas.

Walker et al. (2019) demonstrated how spatial analytical methods can be used to investigate inequalities in access to senior care facilities tailored for specific cultural groups in Vancouver. Their supply/demand analysis enabled the identification of disparities in the availability of such services but as acknowledged by the researchers, used relatively simplistic service ratios of beds per total population for specified cultural groups for census tracts whose geometric centroid was within 2 km walking distance of residential care facilities. Other approaches have integrated GIS functionality and map visualisation with optimisation models to identify the impacts of alternative spatial configurations of residential care facilities and inform policy makers where more homes of sufficient capacity are required to meet potential demand (Cheng & Cui, 2020). The types of approach described in this paper have the potential to build on these approaches, estimating access to residential care homes by including supply‐side (number of beds within residential care homes) and demand‐side (populations within specified age‐cohorts) parameters at more detailed spatial scales.

### 2.2 The use of floating catchment area techniques to measure access to care homes

Floating catchment area (FCA) analysis addresses many of the shortcomings identified in traditional density and proximity scores. Details of the algorithm itself and various adaptations and enhancements of the original methodology are well covered in the published literature so only a summary is presented here (see Langford et al., 2019; Luo & Qi, 2009; Luo & Wang, 2003; McGrail, 2012). The core concept of FCA is the floating catchment, an isochrone constructed around each service demand (and supply) site. This is a polygon centred on the point of interest and representing the area reachable from this point within a stated threshold time/distance.

Floating catchment area permits both proximity of care homes and their potential availability to be incorporated into an accessibility measure. Its output is a ratio expressing service capacity relative to anticipated demand much like the density metrics described above. Although floating catchments are uniquely defined for each demand centre they often overlap, meaning supply points may fall inside multiple catchments. These supply points are shared amongst competing demand centres, so a second calculation (hence its ‘two‐step’ nomenclature) determines the relative share of supply capacity allocated to each competing demand centre. The outcome is a supply/demand ratio computed within the natural activity space of each demand centre that reflects distance‐weighted capacities found in this zone, each normalised by an expected demand placed upon that point of provision.

Floating catchment area methods have appeared in a small number of studies concerned with the provision of residential care homes or home healthcare services. Such studies range from those that applied the original formulation of the technique as developed in the early 2000s to other that include subsequent enhancements (Cai et al., 2017; Cheng, Wang et al., 2012; Ni et al., 2015; Tao & Cheng, 2019; Tao et al., 2014; Wang et al., 2020). Cheng, Wang, et al. (2012) compared nearest distances with a two‐step FCA (2SFCA) approach, to analyse access to residential care resources for over 60 years old in Beijing. Ni et al. (2015) deployed varying catchment sizes by comparing a ‘facility‐to‐population ratio’ to a fixed threshold, and also included a distance‐decay parameter. More recently, Wang et al. (2020) have used 2SFCA to measure bed availability in residential care facilities in Guangzhou and combined this with a detailed road network dataset to draw attention to potential inequalities in provision and possible solutions to address disparities.

Our aim is to investigate the potential of 2SFCA to provide insights into current levels of provision and to establish a benchmark with which to compare future potential closures, changes in the number of beds available, and the impacts of financial pressures on the care home sector immediately post‐COVID and longer‐term, in Wales. To our knowledge, research reported here represents the first attempt to compare FCA techniques with ‘traditional’ metrics to measure potential access to residential or social care homes in the UK context.

## 3 DATA SOURCES

### 3.1 Service supply

**FIGURE 1:**
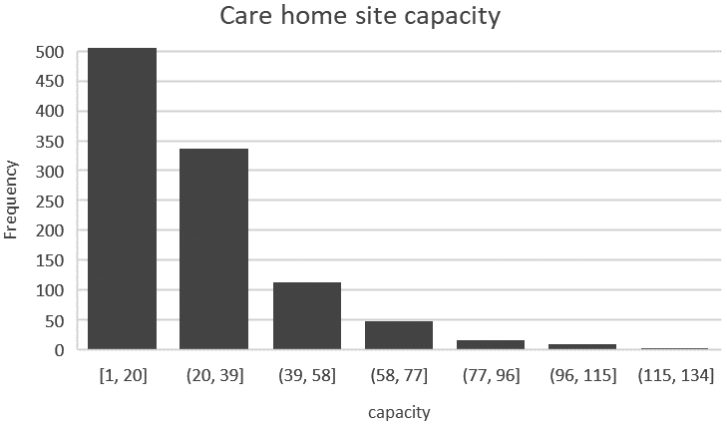
Capacities of care homes in Wales (as of March 2020)

Data concerning care home provision were supplied by Care Inspectorate Wales (CIW). The supply variable relates to beds in adult care home settings regulated by them across Wales; this indicated a total of 25,607 residential and nursing placements were available in March 2020. Only 9% of care home places are provided in local authority run care homes and, as of March 2020, at least three local authorities (of 22) in Wales were reliant on the independent sector for all care home provision (Siôn & Trickey, 2020). Placements were distributed amongst 1069 care home sites, yielding an average of 24 places per home. However, considerable variation exists in individual care home capacity (Figure 1). Many sites are small and over 25% have 6 or fewer.

Two broad categories of care home exist: nursing care homes and residential care homes, with some sites indicating an ability to accept residents with dementia. Detailed patterns regarding the provision of care homes with respect to ownership have been described previously (Public Policy Institute for Wales, 2015). Less than a hundred sites are operated directly by local authorities in Wales, the remainder are privately run. To provide an overall assessment of accessibility to residential homes regardless of provider type, sites both with‐ and without nursing capabilities were included here.

**FIGURE 2:**
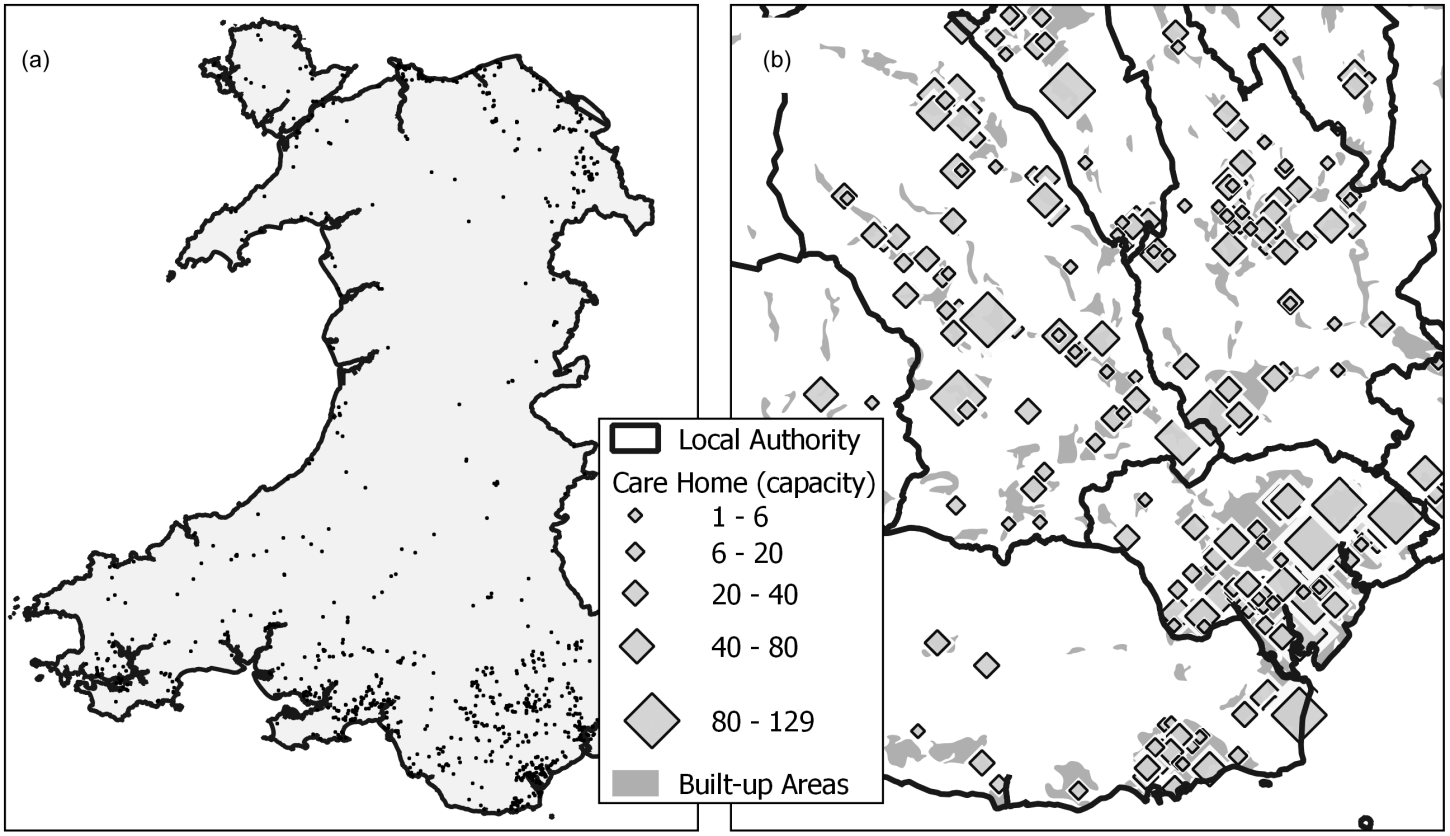
Distribution of care home provision (a) All Wales (b) South‐east Wales

Figure 2 illustrates the distribution of care homes across Wales. Nationally (Figure 2a) this mirrors the broad patterns of population distribution. Locally (Figure 2b) most care homes fall within built‐up areas although their capacities, as stated before, vary considerably.

### 3.2 Service demand

**FIGURE 3:**
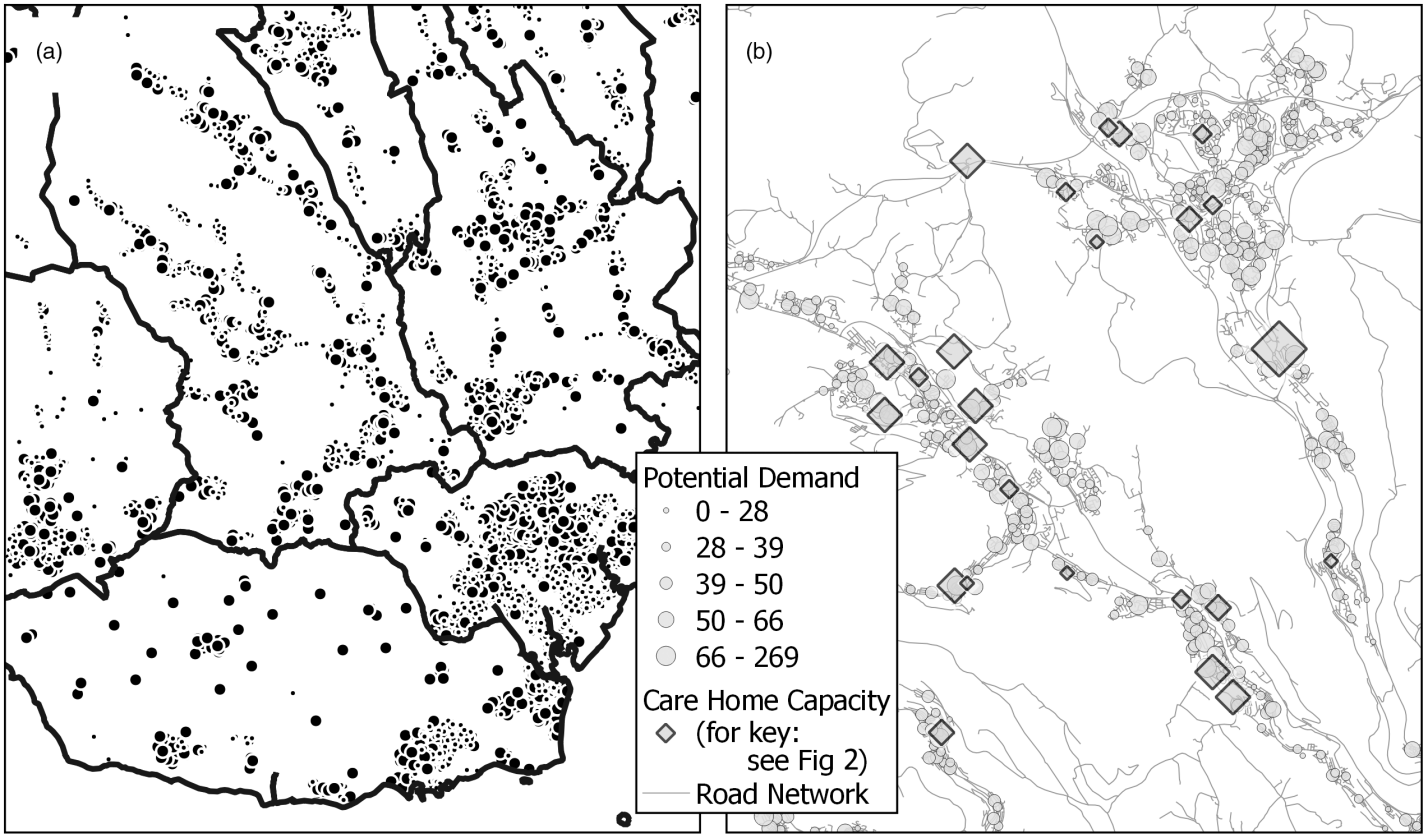
Distribution of potential demand, and example of local supply‐demand complexity (a) South‐east Wales (b) South Wales valleys

Determining the demand placed upon care home services is challenging in several respects. In this study, a simple population‐based approach is adopted. Population counts were obtained for output areas (OAs), the smallest units used for the release of UK census data. The most recently available (March 2019) mid‐year population estimates were used to model service demand (ONS, 2021). The number of residents aged 70 or above was assumed to be indicative of potential demand and assigned to the corresponding population‐weighted centroid. It is recognised that actual or realised demand is likely to be much more complex than this modelled estimate, with a wide range of contributory dependencies such as the health, wealth and other socioeconomic factors of the resident population playing a part. The decision to count residents aged 70 or over is also acknowledged as an arbitrary, if not unreasonable, choice. Notwithstanding these limitations, the resultant dataset provides a detailed spatial breakdown of potential demand as manifested across Wales (Figure 3a).

### 3.3 Network connectivity

The accessibility of care home provision experienced at any specific location in the study area depends upon an intricate interplay of factors, not least the localised balance of supply and demand, and the degree of connectivity via the transport network between residents seeking places and service points providing places. Figure 3b illustrates this complexity, with variable levels of demand expressed at OA centroids needing to be satisfied from irregularly located provision sites with widely varying capacities, separated by travel distances determined through a labyrinthine network of roads. The methodologies that follow explore solutions for assessing geographical variation in care home accessibility arising from this complex situation.

To facilitate route tracing and network distance calculations, a topological street network was constructed for the study area from Ordnance Survey Open Roads© data (OS, 2021) dated July 2020.

## 4 ACCESSIBILITY MAPPING TECHNIQUES AND OUTCOMES

### 4.1 Density analysis

**FIGURE 4:**
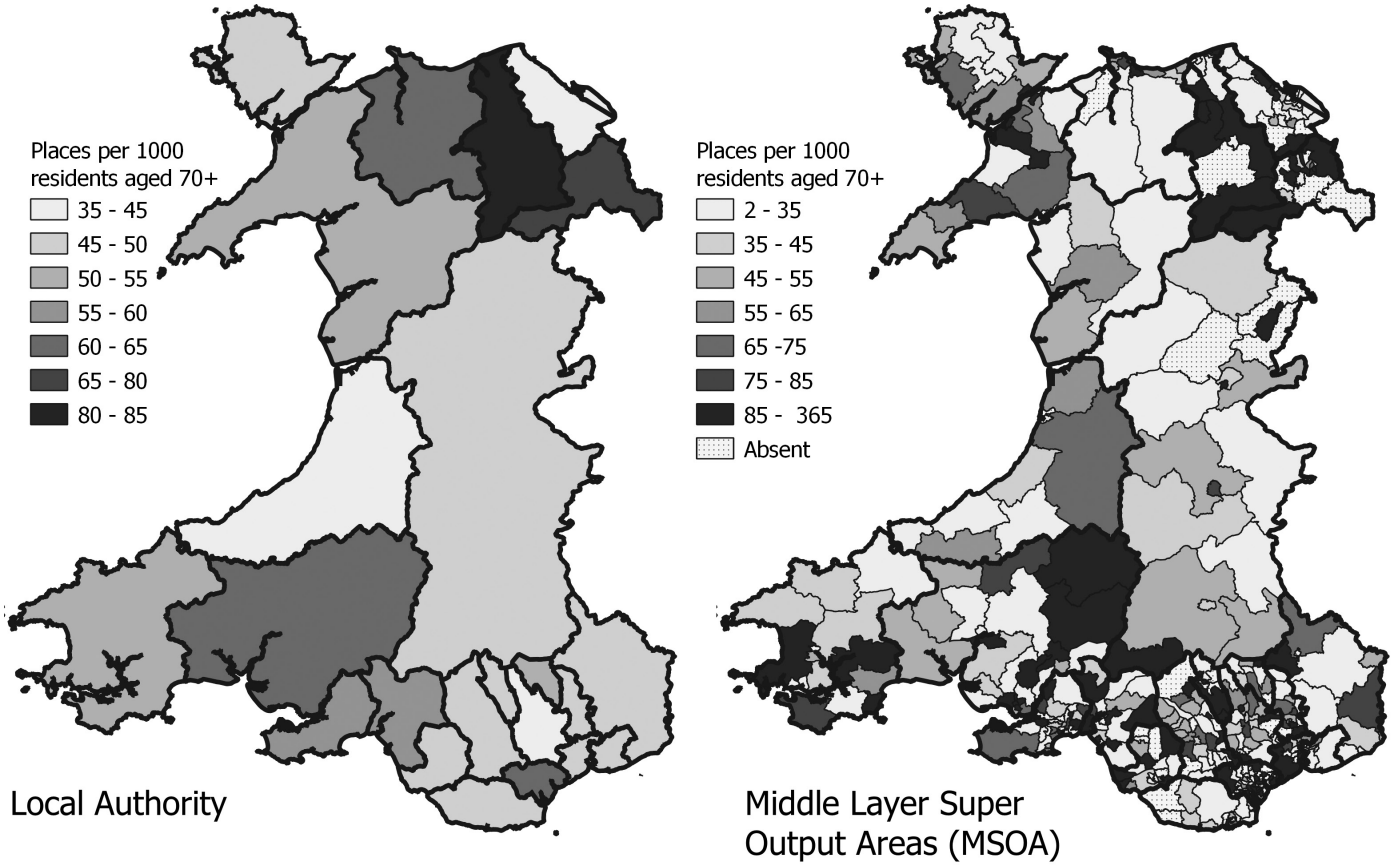
Density measure of accessibility in local authorities and MSOAs. MSOA, middle layer super output area

As stated previously, density metrics are area‐based measures reporting accessibility as a supply–demand ratio. Service capacity in an administrative area is expressed relative to its contained population. Figure 4a illustrates such scores computed for Welsh Local Authorities. These local governance bodies have responsibility for oversight of provision within their region, and for the allocation of places. Residents seeking a care home placement are not legally constrained to utilise services in their local authority area, although in practice this is often the case. We compute a national average of 53.3 places per 1000 aged 70 plus residents, but considerable variation exits between local authorities. This insight may be valuable to those charged with the delivery of care home services in each local authority, but it reveals little about local availability for residents seeking placements. One way to address this shortcoming is to adopt smaller units of analysis. For example, the UK (like most other countries) has a hierarchy of spatial units used to present census information. Local authorities split down into middle layer super output areas (MSOAs), then lower super OAs and finally OAs.

Computing and mapping the density of care home provision at MSOA level (Figure 4b) reveals the extent to which provision varies within local authority areas. However, notable difficulties arise with this solution. First, it is increasingly likely that smaller units contain no care home sites at all, preventing a score from being recorded. Of course, residents can, and most likely will, access provision from nearby areas, so these ‘null’ scores are problematic. Similarly, when using smaller unit of analysis residents are increasingly likely to utilise nearby services falling outside their boundaries, so the relevance of a score computed within its closed borders becomes progressively more questionable. Finally, regardless of unit size adopted this approach assumes equal access to services within itself. In other words, travel distance/time to reach a care home is completely ignored although it is likely to be a key factor in most residents’ perceptions of accessibility to their local services.

### 4.2 Proximity analysis

Given the argument that perceived access to local services is often based upon travel distance/time, many previous studies prefer to adopt proximity‐based measures. These return a score based on the nearest service provision point. Simple approaches use straight‐line distance but increasingly network distance, travel time or even monetary cost, are used as more realistic measures. Using spatial analysis functions provided in a spatial database or GIS, it is possible to compute the proximity of OA centroids to local care homes. In this study, we employ a PostGIS spatial database to store the locations of OA centroids and care homes. Using the OS road dataset and pgRouting library, we compute and then analyse an origin‐destination (OD) matrix of routes between all OAs and their nearby supply points.

**FIGURE 5:**
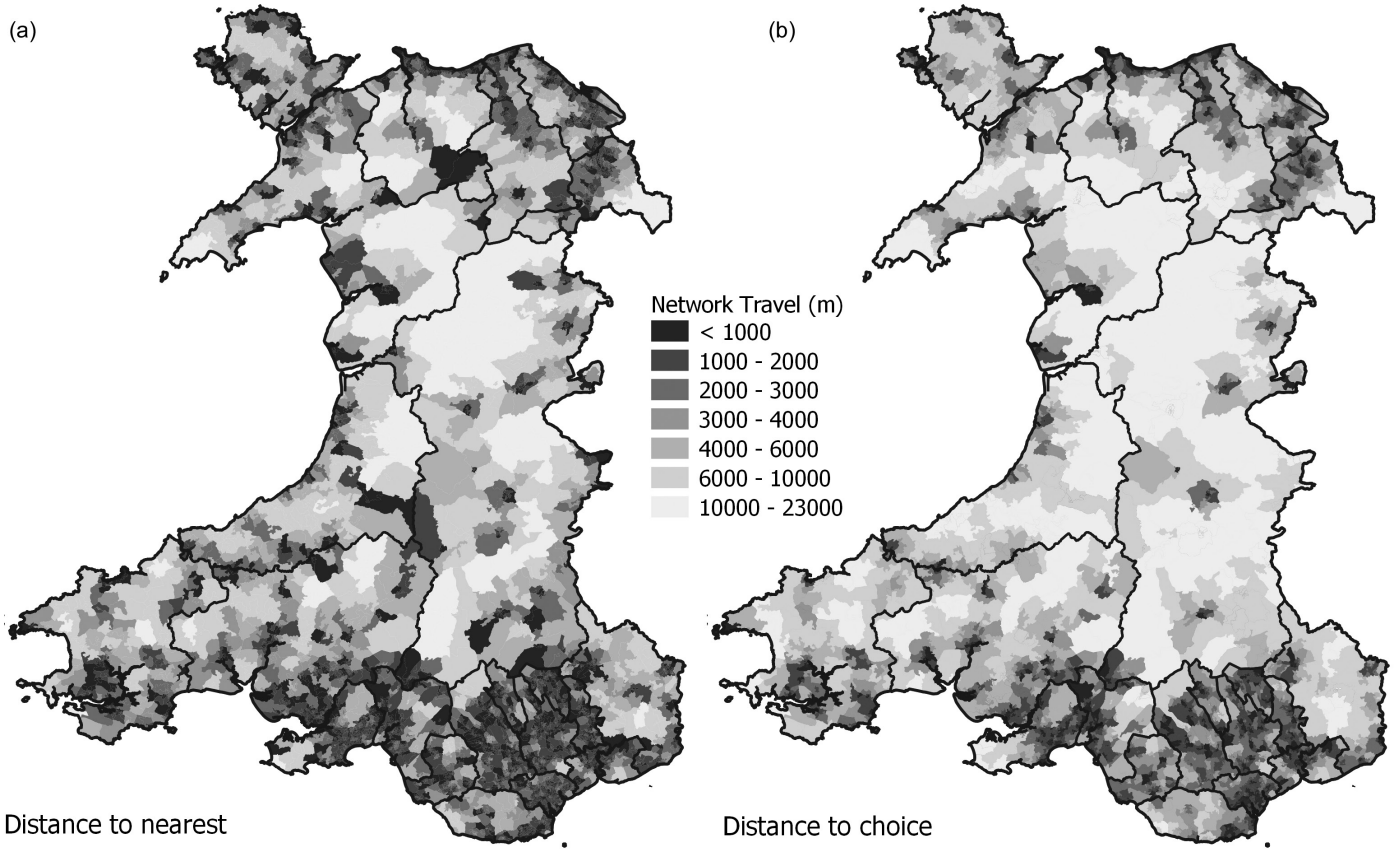
Proximity‐based analyses of care home accessibility (a) Nearest supplier (b) Minimal choice

Figure 5 shows care home accessibility mapped across Wales using simple network distance measures, with the distance to nearest care home reported in Figure 5a. A nearest supply distance is determinable from any point, with this map showing scores for OA centroids. The weakness of this approach is it takes no account of the supply capacity of the nearest site, nor the local demand placed upon it. As reported earlier, many care homes in Wales offer few places so proximity may not reflect closely the likelihood of being able to use the service particularly where demand in the immediate vicinity is high.

Another concern is whether any assessment of accessibility based on a single point of provision adequately reflects broader patterns of provision in the local neighbourhood, or the decision‐making processes of service seekers. For example, it may be expected that sites beyond just the nearest provider might be considered. Travel distance to two or more nearby sites could therefore be a more robust indicator of local availability. Computing a detailed OD matrix lends considerable flexibility to the analyses that may be conducted. In Figure 5b maps travel distance to the second‐nearest care home. This at least represents a measure that incorporates a minimal degree of choice and selection, notwithstanding earlier concerns regarding local capacity and demand. In general, travel distance to the *n*th point of provision increases much more slowly in urban environments than in rural regions.

**FIGURE 6:**
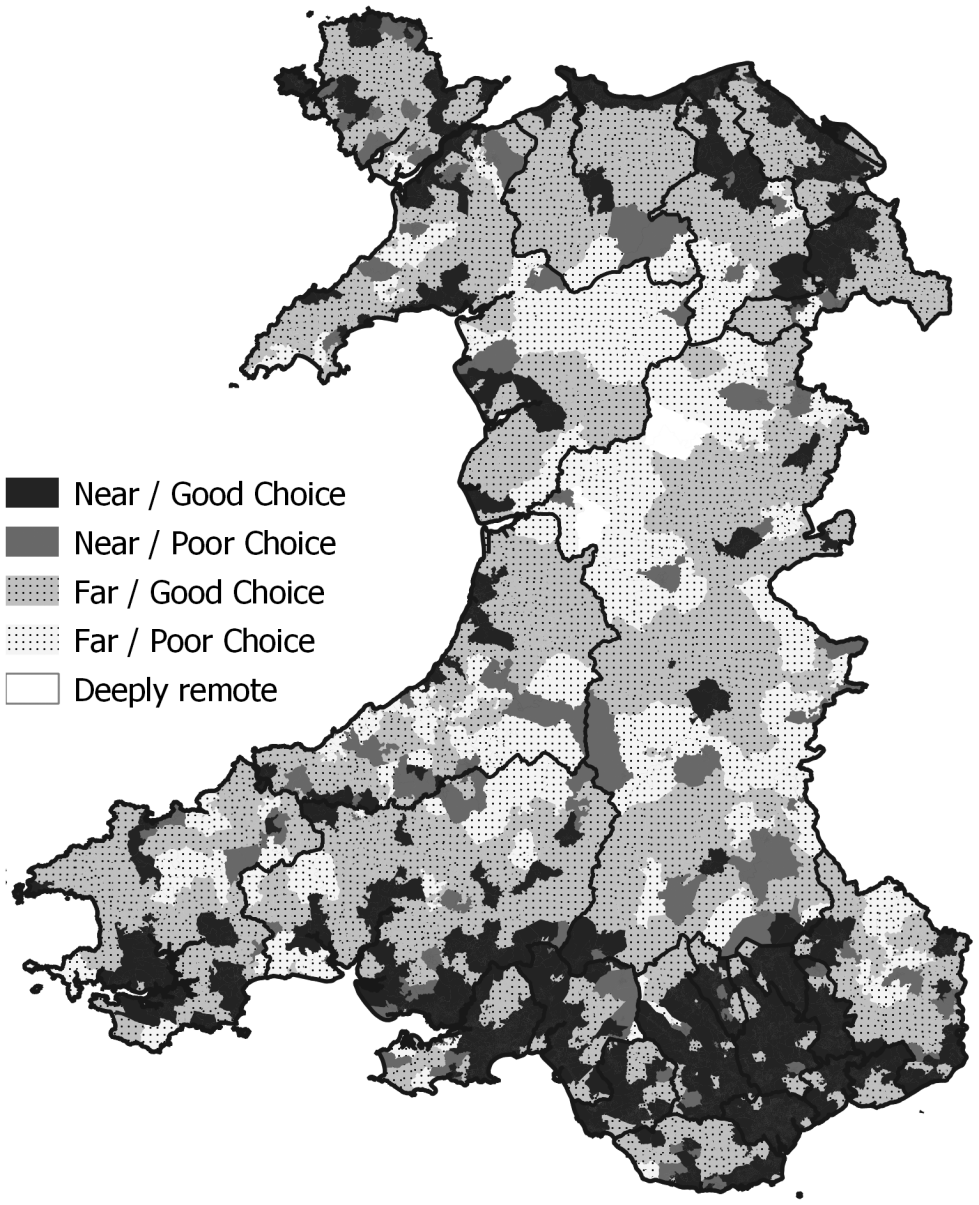
A proximity rule‐based classification of care home accessibility

To further explore the flexibility afforded by the computation of an OD matrix, a rule‐based classification of nearest/next nearest care homes was undertaken (Figure 6). The Near/Good Choice category identifies OAs whose nearest care home is close (<3 km) and where limited further travel (<3 km) is required to reach the next nearest site. Those in the Near/Poor Choice category have a nearby care home but require further travel (>3 km) to reach the next nearest. OAs in the Far/Good Choice category have no nearby care home, but additional travel between nearest and next nearest is small, while in the Far/Poor Choice class the additional travel distance is large. Finally, Deeply Remote locations are located more than 10 km network distance from even their nearest care home.

### 4.3 Floating catchment analysis

**FIGURE 7:**
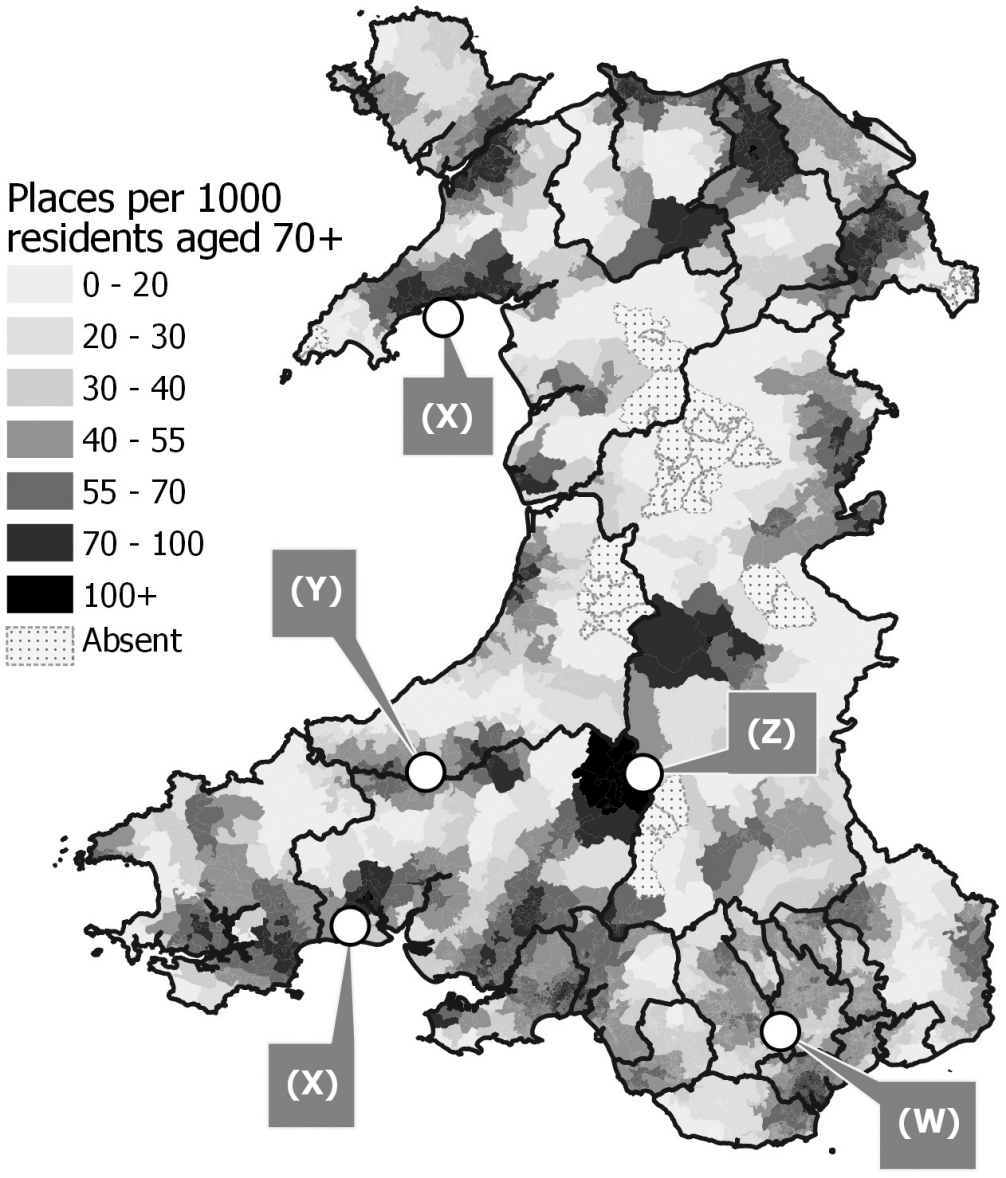
Care home accessibility E2SFCA scores mapped at output area level.

The outcome of an FCA‐based assessment of care home accessibility is presented in Figure 7. Clear differences are evident when visual comparisons are made with density (Figure 4) and proximity (Figure 5) scores, although it is difficult to conduct a detailed analysis using these fixed‐scale static maps; such comparisons are better explored via dynamic web maps whereby a user can alter the scale and map extent at will. OA‐level FCA scores display much finer spatial detail than the MSOA‐level density map. While some OAs are reported as having no access to a care home, this reflects a real absence of supply sites within the chosen travel distance rather than an arbitrary effect of zone boundaries. Furthermore, this issue can be addressed by selecting a larger catchment size, or by adopting other FCA adaptations such as variable‐sized catchments. Accessibility measured through FCA also provides a more sophisticated and nuanced analysis than simple proximity measures. The concentration of care home sites in metropolitan areas and in the heavily populated post‐industrial valleys in the south‐east (label W) is still identified, but the significant demand also present in these areas ameliorates scores towards mid‐range ratios of around 40–60 places per 1000 residents. Several local hotspots with relatively high levels of provision are seen that do not stand out in earlier maps (label X) these arising due to the local balance of supply‐to‐demand. A concentration of higher scores along a major road following the boundary between two LA zones is notably brought out in the FCA analysis (label Y), while the highest service levels are reported in a relatively rural area of mid‐Wales (label Z) and arise mainly due to a small local demand placed upon a modest level of local service supply.

## 5 DISCUSSION

### 5.1 The potential of geographical models for investigating residential and nursing care provision

A key aim of this study has been to provide an analysis of care availability across Wales (as of March 2020), which can be used as a benchmark with which to examine the potential implications of COVID‐19 on the provision of care home places. Figure 7 and the maps provided in the Supplementary Materials illustrate the potential of such models by showing those areas of Wales which appear to have an under‐provision of places (for example parts of the Vale of Glamorgan and Gwent) and those which appear to be well serviced in terms of the balance of supply‐to‐demand (such as pockets of rural areas in North Wales). These models have the potential to contribute to gaining a wider understanding of care home provision in several ways. Firstly, accessibility models can help inform the initial choice of care homes available to residents within specified distance/time thresholds. This in turn can be guided by empirical evidence on average distances between the residents last known address and care homes in different types of geographical contexts (e.g. urban/rural). Secondly, by considering available capacity in relation to potential demand, a factor known to influence decisions concerning choice of home, this analysis can help inform the future planning of service provision and examine whether such facilities are in optimal locations to increase overall accessibility. Thirdly, by considering other supply‐side factors, such as care home quality ratings based on inspections reports, it is possible to refine these models to examine variations in accessibility in relation to those factors likely to impact on choice of provision (such as care home fees and care needs). Fourthly, these types of models can assist longer‐term planning and monitoring of overall care provision by local authorities. As the CMA report (2017; p. 15) highlighted ‘it is essential that there is sufficient capacity of different types of care available in the areas where it is needed’. By examining social care capacity such analyses can contribute to a local assessment of future needs based on projected demand/care requirements within different geographical and social settings.

These types of techniques have been shown to produce different accessibility geographies, with each approach having well‐acknowledged limitations (Neutens et al., 2010). FCA models can address problems such as overcoming the constraints of the ‘container’ type approach which ignore migration across administrative boundaries, and assumptions inherent in proximity approaches by considering all the options available for accessing social care homes within a specified time (or distance) threshold. An additional strength of FCA is the ability to examine the interaction between demand and supply within such thresholds mitigated by the intervening distance between population demand and care home location (i.e. a distance‐decay factor). By accounting for demand based on the characteristics or profiles of population groups more reliant on particular services (e.g., those of a specified age), such demand can be considered in relation to supply‐side capacity constraints (such as capacity or staffing levels of each care home) to provide a more nuanced measure of potential accessibility. This can be further refined in the case of those attending facilities/services by other modes of transport, such as bus and rail services, by using the networking capabilities of GIS to overcome the limitations of a simple Euclidean (straight‐line) analysis, or measures that assume universal access to private (car) transport. Such measures can also be used, alongside socio‐economic data, to examine the impact of access to care homes for delayed transfers of care or on time to admission for patients with conditions such as dementia in rural areas where there may more limited availability or choice of places (Giebel et al., 2021).

### 5.2 Enhancements to the FCA models

Inevitably, assumptions have been made during this study regarding both the time/distance thresholds people are prepared to adopt in their search for an appropriate care home to meet their needs, and the age profile of residents used in the demand‐side of the equation. Regarding the former for example, based on the approach adopted in previous studies, we used a 15‐min drive time to define ‘local’ markets, but catchments can be varied to reflect the actual experience of residents. These models of potential access could be enhanced if empirical evidence was available on distances from last known residence to care home (modelled for car and public transport times) as well as their age on entry. It may also be possible to include distances for visitors since the resident's family will have an important contribution to the decision‐making process and the distance from their home residence could be an equally important factor to take into consideration in the modelling process (Reed et al., 1998).

Further refinements to demand measures should also consider factors such as health needs, especially in relation to requirements for nursing provision within care homes, and the local availability of alternative forms of social care. This would involve a detailed consideration of demographic patterns within Wales to include spatial patterns of disability and deprivation—each of which impacts on demand for care places. On the supply‐side more information on the nature of care home provision could also be incorporated here to examine how a measure of quality of care (as evidenced through inspection reports from regulation authorities such as the Care Quality Commission in England or Care Inspectorate Wales) can be used to examine the choice of residential care homes of a certain quality within reasonable geographic distance thresholds.

The number of beds has been used as the supply‐side measure, but we have no data on occupancy rates within individual care homes, which Siôn & Trickey, 2020 suggest fluctuates around 90% in Wales. In addition, an Incisive Health (2019) report drew attention to the undersupply of nursing home beds in England (as well as an acute shortage of nurses). There may well be regional variations in the demand for care services which reflect variations in for example those needing nursing care for a range of disabilities that could help refine these models. The data we have provide an aggregate number of beds for residential and nursing beds. If data could be split into individual supply‐side capacities of both residential and nursing places available at specified locations, the models could be tailored to investigate trends in potential accessibility that acknowledges variations in potential demand.

Finally, our models are predicated on the assumption of equal access to all residents of an area regardless of financial means of accessing care. The financial circumstances of individuals and families are a material factor influencing choice of care home. Charging for social care varies by country—there are variations across the four countries of the UK. In Wales, if people have capital (including in many cases the value of their home) of over £50,000 they may have to pay the full cost of their residential care. If people have capital at or below this limit, the local authority will help to offset the costs of residential care. Those who can afford to fund their own care can choose any residential home which meets their needs and can accept them. However, those who receive public funding are limited to care homes whose fees are within the range that their local authority is willing to pay, unless their family can top up the payment that the local authority makes. Previous studies have drawn attention to the need to consider financial forces impacting on access to residential care home places (Vaňková & Vavrek, 2021) and the accessibility implications of such factors could form the basis of our future research into wider aspects of care home provision.

## 6 CONCLUSIONS AND POLICY IMPLICATIONS

Even before the outbreak of COVID‐19, there were well documented pressures in the social care sector arising from projected rise in demand for care and the increasing need to care for those with complex health needs. The Competition and Markets Authority (2017) suggested that between 2015 and 2025, the UK care home population was projected to grow annually by between 1.4% and 2.9% and could reach half a million by 2025. In Wales, the number of people aged 65 or over receiving residential services is set to increase to just under 30,000 by 2035 from just over 11,000 in 2015 (Welsh Government, 2019). A recent Welsh Government White Paper (2021) estimated the number of people aged 65 and over is projected to rise to one in four of the population by 2050 and that many in the cohort may have complex health needs with a predicted doubling of older adults living with severe dementia predicted to just under 54,000 by 2040.

Local authorities, working in partnership with Local Health Boards (LHBs) and other organisations, are responsible for social care in their areas, for examining existing provision close to their residents’ homes, families and communities, and for planning levels of resources needed to meet future demand. As highlighted in the CMA report (2017; p. 87) they ‘must also publish a local market stability report which must include an assessment of the sufficiency of the provision of care and support in the area’. The aims of this study have been to measure the current level of residential care home provision at detailed spatial scales and to demonstrate how geographical modelling tools have the potential to identify variations in the provision of care home places. As well as being of use to such organisations, care home providers could also benefit from approaches that help guide the types of provision needed to meet local community needs and respond to demographic trends. The CMA (2017) report drew attention to the need for neighbouring authorities to consider the overall availability of care home services instead of narrowly focusing on provision within their local area boundaries. In this paper, we have demonstrated how advanced spatial analytical techniques enable wider assessments to be made that include a consideration of such ‘out of county’ placement opportunities.

We suggest that the outputs from this analysis provide an important benchmark with which to analyse the impacts of future developments in the provision of residential care homes in Wales on the access scores presented here. Data used here relate to the situation in March 2020 and thus largely pre‐date the pandemic. On the supply‐side, there will be inevitable impacts on financial budgets within local authorities which could impact on the profitability of some care home providers who have seen occupancy levels decline during the last year due to COVID‐19 and which, a recent NAO report estimates, may take some providers 18 months to address (National Audit Office, 2021). These may well interact with long‐standing demographic and health trends and financial pressures outlined earlier that were the focus of much of the literature in the years leading up to the pandemic. Further research is needed to examine the implications of such trends for the types of access measures developed in this study.

## CONFLICT OF INTEREST

No potential conflicts of interest to declare.

## Supporting information

Supplementary Material

## Data Availability

Data on provision were made available for March 2020 by Care Inspectorate Wales.
