# Supplementary material for: Towards an understanding of inequalities in accessing residential and nursing home provision: The role of geographical approaches
Source: Health Soc Care Community. 2022 Feb 25;30(6):2218–29. doi: 10.1111/hsc.13770 (PMC10078699; doi:10.1111/hsc.13770)
Supplement: Supplementary file 1 — Supplementary Material [file HSC-30-2218-s001.docx]

**Exploring the sensitivity of results to service demand evaluations.**

The determination of demand placed upon care home services is challenging. The analysis conducted in the main text adopted the number of Output Area (OA) residents aged 70 or above as potential demand. This was acknowledged as a somewhat arbitrary choice, and these supplementary maps explore the sensitivity of results to this modelling parameter.

Supplementary Map 1 shows (left) how demand population inevitably declines as an older population base is selected, but also how geographical variation exists amongst OA centroids in the precise rate of reduction. Two-step floating catchment area scores are a supply-demand ratio, so with a constant supply capacity (care home places) but lower demand population, scores rise as a more aged population base is used. This necessitates careful adjustment of class boundaries to allow meaningful comparisons between maps, as further discussed below. Although the broad distribution of accessibility levels appears to be quite stable (see Map2), localised examples were found where some sensitivity was apparent. Shown on the right is the area between Pwllheli and Criccieth on the Llyn Peninsula, where accessibility declines in relative terms as a more aged population base is adopted.

Supplementary Map 2 shows equivalent outcomes to those presented in the main text as Figure 7, based upon aged 75 and over (left), and aged 80 and over (right) populations. Wales-wide the populations in these age brackets are 480,234 (70+), 300,948 (75+), and 174,163 (80+). To account for the effect this has on resultant supply-demand ratios the class boundaries of these maps are adjusted by the national proportional scale difference using the original aged 70+ as the baseline (specifically, a factor of 1.59 and 2.75 respectively). A visual comparison confirms that the broad patterns of reported high and low accessibility remain stable and consistent between these various alternative modelling scenarios.


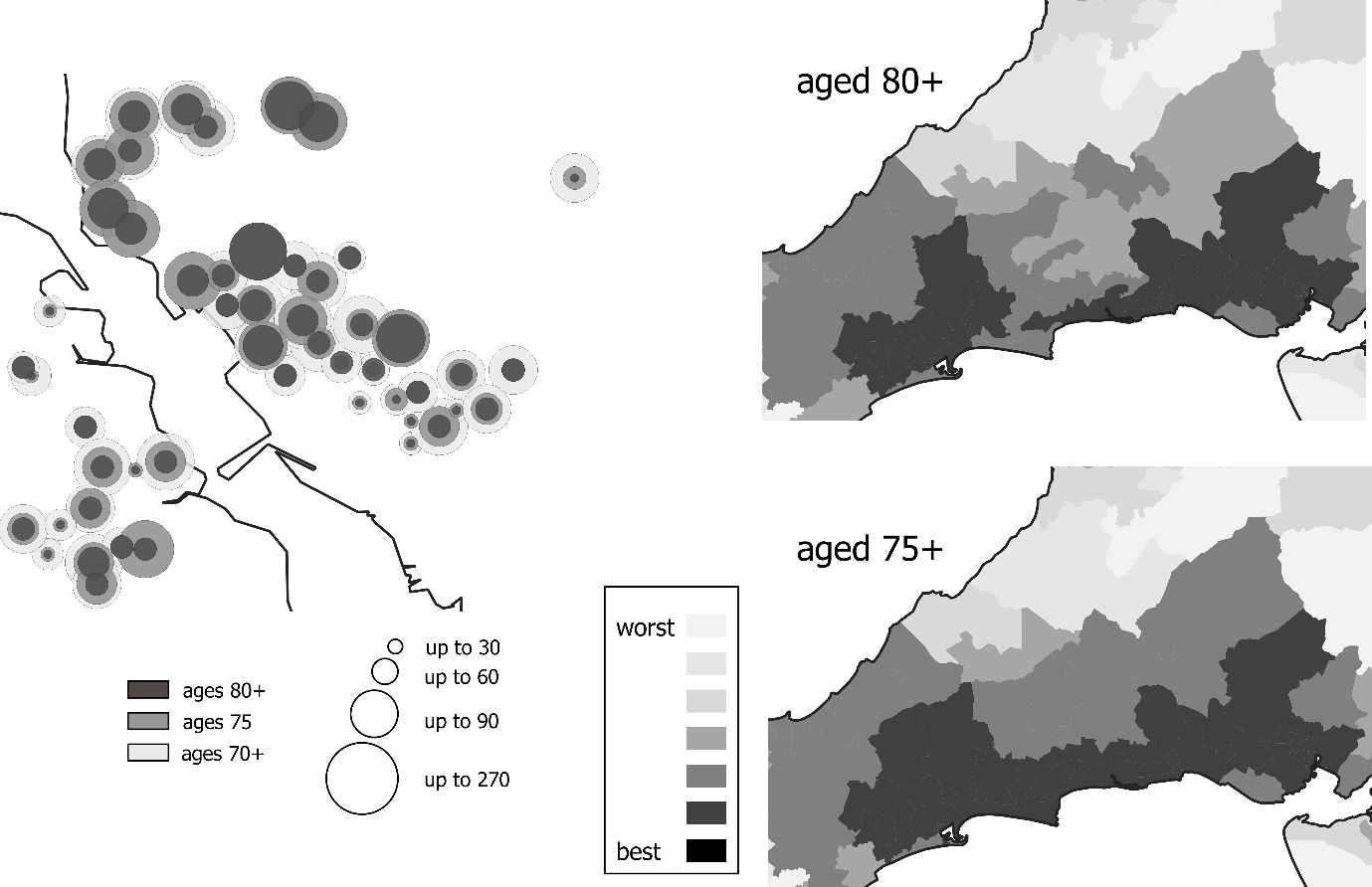
Supplementary Map 1: Population demand at Output Area level using alternative age groups (left), and localised effect on resultant floating catchment area accessibility scores (right).


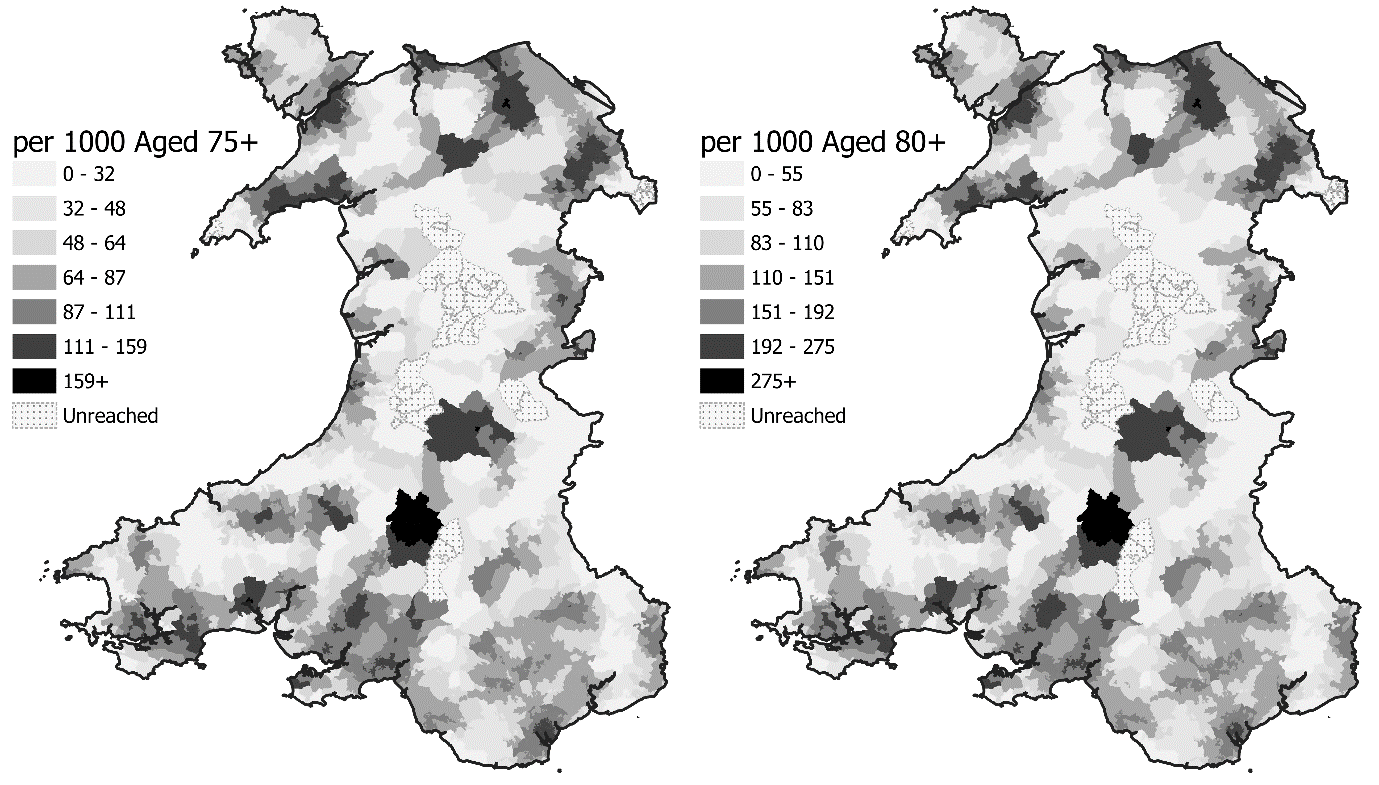


Supplementary Map 2: Care home accessibility E2SFCA scores mapped at Output Area level using aged 75 and over (left), and aged 80 and over (right) as demand population estimates.
